# Insights into the potential for mutualistic and harmful host–microbe interactions affecting brown alga freshwater acclimation

**DOI:** 10.1111/mec.16766

**Published:** 2022-11-23

**Authors:** Hetty KleinJan, Clémence Frioux, Gianmaria Califano, Méziane Aite, Enora Fremy, Elham Karimi, Erwan Corre, Thomas Wichard, Anne Siegel, Catherine Boyen, Simon M. Dittami

**Affiliations:** ^1^ Station Biologique de Roscoff, Laboratory of Integrative Biology of Marine Models Sorbonne University, CNRS Roscoff France; ^2^ CEBEDEAU, Research and Expertise Centre for Water Quartier Polytech 1 Liège Belgium; ^3^ Inria, CNRS, IRISA University of Rennes Rennes France; ^4^ Inria University of Bordeaux, INRAE Talence France; ^5^ Institute for Inorganic and Analytical Chemistry Friedrich Schiller University Jena Jena Germany; ^6^ Station Biologique FR2424, ABiMS, Sorbonne Université, CNRS Roscoff France

**Keywords:** brown algae, holobiont, host–microbiome interaction, low salinity acclimation, metabolic networks, metabolite profiling, meta‐transcriptomics, virome

## Abstract

Microbes can modify their hosts' stress tolerance, thus potentially enhancing their ecological range. An example of such interactions is *Ectocarpus subulatus*, one of the few freshwater‐tolerant brown algae. This tolerance is partially due to its (un)cultivated microbiome. We investigated this phenomenon by modifying the microbiome of laboratory‐grown *E. subulatus* using mild antibiotic treatments, which affected its ability to grow in low salinity. Low salinity acclimation of these algal‐bacterial associations was then compared. Salinity significantly impacted bacterial and viral gene expression, albeit in different ways across algal‐bacterial communities. In contrast, gene expression of the host and metabolite profiles were affected almost exclusively in the freshwater‐intolerant algal‐bacterial communities. We found no evidence of bacterial protein production that would directly improve algal stress tolerance. However, vitamin K synthesis is one possible bacterial service missing specifically in freshwater‐intolerant cultures in low salinity. In this condition, we also observed a relative increase in bacterial transcriptomic activity and the induction of microbial genes involved in the biosynthesis of the autoinducer AI‐1, a quorum‐sensing regulator. This could have resulted in dysbiosis by causing a shift in bacterial behaviour in the intolerant algal‐bacterial community. Together, these results provide two promising hypotheses to be examined by future targeted experiments. Although they apply only to the specific study system, they offer an example of how bacteria may impact their host's stress response.

## 1 INTRODUCTION

Host–microbe interactions are known to play essential roles in the lives of virtually all organisms. Among the best‐known examples are the animal intestinal microbiota (Shreiner et al., 2015) and the plant rhizosphere (Ofek et al., 2006), where microbes have been shown to influence their hosts' stress tolerance and play critical roles in host health and nutrition (e.g., de Zelicourt et al., 2013; Mendes et al., 2013). Recently, more examples of similar interactions are being studied in all domains of life, including in marine environments and algae, where microbes are actively gardened by their host (Kessler et al., 2018; Saha & Weinberger, 2019) and may impact, for instance, algal defences (Saha et al., 2018) or morphology (Wichard, 2015).

Brown algae are multicellular members of the stramenopile lineage and frequently form the dominant vegetation in intertidal zones of temperate marine coastal ecosystems (Wahl et al., 2015). They are essential as ecosystem engineers and increasingly being exploited as a food source (Food and Agriculture Organization of the United Nations, 2016) and for the production of alginate (McHugh, 2003) or other high‐value compounds (Milledge et al., 2016; Silva et al., 2020). Like the examples mentioned above, brown algae have formed tight relationships with their associated microbiota, which may provide them, for instance, with vitamins, phytohormones, and protection against disease or fouling (Egan & Gardiner, 2016; Goecke et al., 2010; Karimi et al., 2020; Wahl et al., 2012).

*Ectocarpus* is a cosmopolitan genus of small filamentous brown algae that is easy to cultivate in the laboratory and is closely related to large kelp‐forming brown algal species. The *Ectocarpus* sp. strain Ec32 has been established as a model system to study brown algal biology (Charrier et al., 2008), and its genome was the first brown algal genome to be published (Cock et al., 2010). *Ectocarpus* is furthermore a model to study algal bacterial interactions, with studies demonstrating, for instance, its reliance on bacteria‐produced cytokinins, which serve as morphogens for the alga (Pedersén, 1968, 1973; Tapia et al., 2016).

One species of *Ectocarpus*, *Ectocarpus subulatus*, is of particular interest, as it has been described in river habitats (Dittami, Peters, et al., 2020b; West & Kraft, 1996), yet its capacity to tolerate freshwater medium (salinity 1.6) in the laboratory seems to be conditioned by its microbiota (Dittami et al., 2016): antibiotic‐treated cultures still grew in seawater medium but no longer in freshwater medium. However, growth could be restored by restoring the microbiome with medium from an untreated culture. We speculate that this dependence on bacteria may be related to (1) the direct production of compounds such as osmolytes or chaperones that enhance the algal capacity to grow in low salinity, (2) general bacterial services that are required for algal growth regardless of the salinity, (3) protective functions of some bacteria to prevent phenomena of dysbiosis, or (4) a combination of these effects. However, despite extensive cultivation efforts, including dilution to extinction techniques and a range of different culture media (KleinJan et al., 2017), neither the bacteria responsible for this phenomenon nor the underlying bacterial functions have been identified. This could be because the underlying interactions are complex and involve numerous actors or because the bacteria of interest are uncultivable. *E. subulatus* constitutes an example where host–microbiome interactions impact the properties of a biological system, but the complexity of the interactions and the system prevents us from isolating a single responsible microbe or microbial function.

The role of *Ectocarpus*‐associated bacteria in the freshwater response was investigated in this study using a microbiome‐modulated approach. Rather than starting from a collection of cultured bacteria, we worked with algae, including their natural microbiome in culture, and then treated these algae with various antibiotic combinations. These antibiotics were powerful enough to change the algal microbiome but not completely eliminate it. Several of the treated algae were able to survive in fresh water. We then used a combination of metagenomics, meta‐transcriptomics, and metabolomics to investigate *E. subulatus* with three distinct microbial communities (MC1–MC3), each with its own response to fresh water. This approach allowed us to develop testable hypotheses about how these algal–bacterial communities respond to low salinity and, in particular, how variation in initial microbiome composition correlates with acclimation capacity. Although these findings cannot be generalized beyond the boundaries of this model system, they constitute a valuable example of how host–microbe interactions may impact stress tolerance.

## 2 MATERIALS AND METHODS

### 2.1 Preparation of algal cultures with modified microbiomes

Starter cultures of the *Ectocarpus subulatus* freshwater strain (EC371, accession CCAP 1310/196, West & Kraft, 1996) were grown in 90 mm Petri dishes in natural seawater (NSW; salinity 35 ppt, collected at Roscoff 48°46′40″N, 3°56′15″W, 0.45 μm‐filtered, autoclaved at 120°C for 20 min), enriched with Provasoli nutrients (2 mg/L Na_2_EDTA, 2.24 mg/L H_3_BO_3_, 240 μg/L MnSO_4_·H_2_O, 44 μg/L ZnSO_4_·7 H_2_O, 10 μg/L CoSO_4_·7H_2_O, 0.7 mg/L FeEDTA, 0.6 mg/L Na_2_EDTA, 4 mg/L Na_2_ β‐glycerol PO_4_·5H_2_O, 35 mg/L NaNO_3_, 0.875 μg/L vitamin B_12_, 40 μg/L thiamine, 4 μg/L biotin; Starr & Zeikus, 1993). They were kept under our laboratory's standard algal culture conditions at 13°C with a 12 h dark–light cycle (photon flux density 20 μmol/m^2^/s). Algal starter cultures were then treated with different antibiotics generating different microbial communities, and their capacity to grow in diluted natural seawater medium (5% NSW, 95% distilled water) was assessed (Table S1). Based on the results of these experiments, algae with three microbial communities were selected for the final acclimation experiments. Two microbial communities (MC1 and MC2) were selected to provide two different examples of freshwater‐tolerant algal‐bacterial associations. They were obtained after short (3 days) treatments with antibiotic solutions (rifampicin, penicillin, neomycin, each at 100 μg/mL for MC1; the same as before with additional streptomycin 25 μg/mL, and chloramphenicol 5 μg/mL for MC2). As described in the Results, algae with these communities could grow in diluted natural seawater medium. The third microbial community (MC3) was selected as an example of freshwater‐intolerant algal‐bacterial associations. The treatment used to obtain algae with MC3 was chosen to keep the data comparable with our previous study (Dittami et al., 2016): algal filaments were placed in the proximity of penicillin (12,000 IU), chloramphenicol (0.75 μg), polymyxin B (0.75 μg), and neomycin (0.75 μg) discs on Petri dishes with Zobell agar for 5 weeks. As expected, algae with this microbial community were no longer freshwater‐tolerant.

To ensure the availability of sufficient viable growing algal biomass, algae and their microbial communities were cultivated in antibiotic‐free 100% NSW for 2 weeks (algae with MC1 and MC2) or 5 months (algae with MC3; in this case the medium was renewed monthly). Although the algae had undergone different recovery treatments, they were comparable in growth and biomass at this point. Then a few filaments each (c. 5 mm) with three different microbial communities were transferred to 10 L culture flasks with Provasoli‐enriched NSW. They were grown under identical conditions in antibiotic‐free Provasoli‐enriched NSW with monthly medium changes for approximately 2 months to obtain sufficient biomass.

**FIGURE 1:**
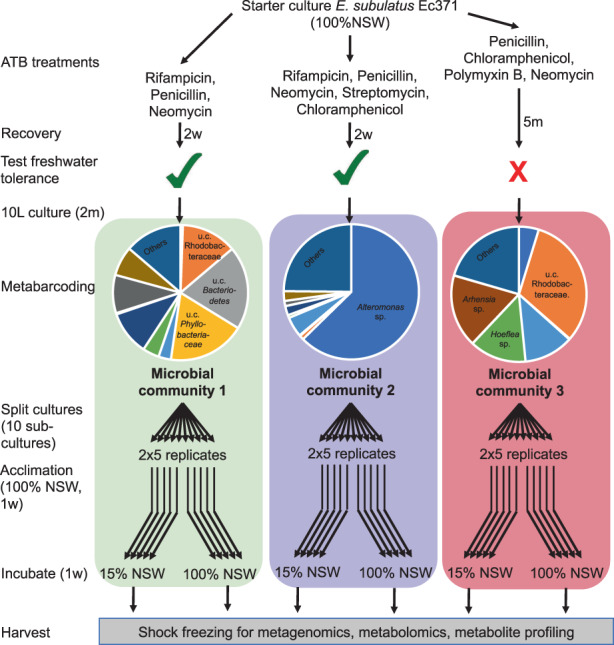
Overview of the experimental setup comprising three different microbial communities and two salinity conditions. All conditions were derived from the same starter culture, but antibiotic (ATB) treatments were carried out with three different ATB mixes (see Section 2), leaving hosts with different microbial communities and different salinity tolerance, termed “microbial communities (MC) 1–3”. Pie charts in the metabarcoding part show the ten most abundant genera in each microbial community. The low‐salinity response of 5 replicate algae with each of these communities was examined. 100% NSW, natural seawater; 15% NSW, 15% NSW in distilled H_2_O (volume/volume); m, month(s); w, week(s)

To ensure that the algal cultures differed in their associated microbial community composition and before acclimation experiments (see below), two samples of each culture (technical replicates) were used for 16 S rRNA gene metabarcoding. The differences observed at this point by the metabarcoding analysis were later confirmed with the metatranscriptome data examining the normalized transcriptional activity per bin. Then cultures were split into 10 replicate cultures in 2 L culture flasks, each replicate consisting of similar microbial communities and kept in NSW medium until the start of the acclimation experiment. The experimental setup is shown in Figure 1.

### 2.2 16 S rRNA gene metabarcoding

16 S rRNA metabarcoding was carried out as previously described (KleinJan et al., 2017). Briefly, total DNA was isolated (NucleoSpin Plant II, Machery‐Nagel; standard protocol) from snap‐frozen tissue and purified with Clontech CHROMA SPIN‐1000 + DEPC‐H2O columns. The V3–V4 region of the 16S rRNA gene was amplified and sequenced with Illumina MiSeq technology by MWG Eurofins Biotech (Ebersberg, Germany) using their proprietary protocol and yielding 1,859,076 reads. After quality trimming using the FASTX Toolkit (quality threshold 25; minimum read length 200), data were analysed with Mothur (version 1.38.0) according to the MiSeq Standard Operating Procedures (Kozich et al., 2013). Sequences were aligned to the nonredundant Silva SSU reference database version 123 (Quast et al., 2013), chimeric sequences removed using Uchime (Edgar et al., 2011), clustered into operational taxonomic units (OTUs) at a 97% identity level, and classified taxonomically (Wang et al., 2007).

### 2.3 Acclimation experiments

Acclimation experiments were carried out to elucidate differences in the transcriptomic and metabolic response to low salinity of algae associated with the three different microbial communities. Because natural seawater medium diluted to 5% NSW was lethal to algae with MC3 (Table S1, complete discoloration of algal filaments after 3 weeks), we opted for a final concentration of 15% NSW and 85% distilled water enriched with Provasoli nutrients. This corresponds to a total salinity of 5.1 and thus is within the range of salinities found in the Hopkins River at locations with *E. subulatus* populations (salinity 2–6) (Dittami, Peters, et al., 2020b). At 15% NSW, growth of algae with MC3 was inhibited – that is, unlike in tolerant cultures, we did not visually detect algal growth for 3 weeks, but the condition was not lethal (i.e., filaments still had their typical brown colour, and started to grow again when moved back to NSW medium). Algae from five replicate cultures with each microbial community (prepared as described above) were collected using UV‐sterilized coffee filters, transferred from 100% NSW to new 2 L flasks with fresh 15% NSW, and the other five replicates were transferred to fresh 100% NSW as a control. Cultures were left to acclimate to these conditions for 1 week – that is, the time needed to observe growth in the tolerant cultures. Then algal tissue was collected again using UV‐sterilized coffee filters. Coffee filters and all nondisposable equipment used below, as well as the working environment, were treated with RNaseZAP (Sigma‐Aldrich) before use. Excess water was removed by drying with a clean paper towel, and tissues were snap‐frozen in liquid nitrogen and stored at −80°C until further processing.

### 2.4 Metagenome and metatranscriptome sequencing

Approximately 50 mg (fresh weight) of algal tissue was ground in liquid nitrogen and sterilized sand using a pestle and mortar. Nucleic acids were extracted as previously described by Le Bail et al. (2008) using a CTAB‐based lysis buffer and phenol‐chloroform purification. RNA and DNA were separated after precipitation with LiCl (0.25 volumes, 12 M, overnight, −20°C); the RNA was resuspended in 300 μl RNase‐free water and purified with 1 volume of phenol: chloroform (1:1, pH 4.3, 20 min, 4°C) and twice with one volume of chloroform. It was then precipitated (0.1 volumes 3 M sodium acetate, 3 volumes 100% ethanol, 2 h at −80°C), washed with ice‐cold 70% ethanol, and resuspended in 15 μl RNase free water. Ribosomal RNA molecules were depleted from the RNA extracts using the RiboMinus Plant Kit for RNA‐Seq (ThermoFisher Scientific). This allowed selectively removing abundant nuclear, mitochondrial, and chloroplast rRNAs of the algal host. Further, 1 μl of the bacterial probe (RiboMinus transcriptome isolation kit for yeast and bacteria, ThermoFisher Scientific) was added in the last 5 min of hybridization to remove bacterial ribosomal RNA. The RNA was concentrated (RiboMinus Concentration module; ThermoFisher Scientific) before checking the quality with a bioanalyser (Agilent). Library preparation (TruSeq Stranded mRNA Library Prep kit; Illumina) and sequencing (five lanes, 150 bp read length, paired‐end, Illumina HiSeq 3000) were carried out for four of the five replicates per condition.

DNA was extracted from the supernatant of the LiCl precipitation. It was precipitated with one volume of isopropanol, resuspended in 300 μl DNA‐free water, and purified once with one volume of phenol:chloroform:isoamylic alcohol (25:24:1; pH 8) and twice with one volume of chloroform. Finally, the DNA was precipitated (3 volumes of 100% EtOH +0.1 volumes of 3 M sodium acetate) and resuspended in 50 μl of molecular biology grade water. DNA extracts from all samples were pooled (same concentration of each sample), and the pooled DNA was purified using caesium chloride gradient centrifugation (Le Bail et al., 2008). The Illumina TruSeq DNA Nano kit was used to create a single library, which was then sequenced on four lanes of Illumina HiSeq 3000 (2 × 150 bp read length, paired‐end, GeT PlaGe Genotoul platform).

### 2.5 Metagenome analyses

Raw sequencing reads were quality‐trimmed with Trimmomatic (version 0.36, minimal Phred score: 20, minimal read length: 36 nucleotides) (Bolger et al., 2014) and aligned to the *E. subulatus* Bft15b reference genome (Dittami, Corre, et al., 2020a) using star aligner version 2.6.0a (Dobin et al., 2013) to remove algal reads. Nonaligning (i.e., mainly bacterial) reads were then assembled using metaspades version 3.11.0 (Nurk et al., 2017) using default parameters except the memory limit, which was set to 950 GB and the “‐‐careful” option. The resulting contigs were filtered by length (>500 bp), and the remaining *Ectocarpus* contigs were removed with taxoblast version 1.21 (Dittami & Corre, 2017). Metagenomic binning was carried out using anvi'o version 4.0 according to the “anvi'o User Tutorial for Metagenomic Workflow” (Eren et al., 2015): Raw metagenome reads were mapped against the contig database using bwa‐mem (version 0.7.15), and taxonomy was assigned to the contigs using centrifuge (Kim et al., 2016) and the NCBI nucleotide nonredundant database (nt_2018_3_3). Finally, contigs were clustered according to GC content and coverage, and the anvi'o interactive interface was used to curate the bins manually. The quality of the metagenomes was assessed based on the abundance of single‐copy core genes (SCGs; Campbell et al., 2013). One bin containing essentially *Ectocarpus* reads that had been missed during the previous cleaning steps was manually removed at this stage. The remaining bins were bacterial and annotated with prokka version 1.13 (Seemann, 2014).

Metabolic networks were reconstructed using Pathway Tools version 20.5 (Karp et al., 2016) and the scripts included in the AuReMe pipeline (Aite et al., 2018). This data set served as a backbone for analysing bacterial gene expression from the metatranscriptomic data.

### 2.6 (Meta)transcriptome analyses

Ribosomal RNA reads that remained despite the RiboMinus treatment were removed in silico using sortmerna version 2.1 (Kopylova et al., 2012) and default parameters. After quality trimming (trimmomatic 0.36, minimal Phred score: 20, minimal read length: 36 nucleotides), reads were mapped first to the *E. subulatus* Bft15b reference genome for algal gene expression analysis, and the remaining reads to the metagenomic bins generated as described above using star aligner version 2.6.0a. The ratio of bacterial to algal mRNA was compared across samples using a one‐way ANOVA followed by Tukey's HSD test using past version 4 (Hammer et al., 2001). Then, both the algal transcriptome and the bacterial metatranscriptome were analysed separately. In both cases, deseq2 (Love et al., 2014) was used for principal component analysis (PCA, rlog‐transformed data) and for the detection of significantly differentially expressed genes (DEGs). However, read coverage was insufficient for the bacterial metatranscriptome to conduct differential gene expression analysis on a bin‐per‐bin basis. Therefore, we summed up the bacterial expression data associated with the same metabolic reaction across the different metabolic networks. This overall transcription of a metabolic function within the entire microbiome was used to identify differentially expressed microbial reactions in the same way as the algal DEGs.

The following comparisons were carried out for the algal transcriptome and the bacterial metabolic reactions. First, for algal cultures with each of the three microbial communities (MC1, MC2, MC3), we individually determined genes and reactions that were differentially expressed in 15% NSW compared to 100% NSW. Then, the same salinity treatments of cultures with the microbial communities yielding freshwater‐tolerant algae were grouped (i.e., MC1–100% and MC2–100% as well as MC1–15% and MC2–15%). Algae with these two microbial communities were compared to the algae with a microbial community that did not enable growth in fresh water (MC3–100% and MC3–15%). For this comparison, the statistical design considered both factors (microbial community and salinity) and the interaction term. Lastly, we directly compared the gene expression profiles between the two freshwater‐tolerant algal/bacterial communities, algae with MC1 and algae with MC2, keeping salinity as a cofactor. All genes or reactions with an adjusted *p*‐value < .05 and a fold change in expression >1.5 were considered significantly differentially expressed. Gene set enrichment analyses were performed for sets of differentially expressed genes using Fisher's exact module within blast2go (version 4.1.9; 2‐tailed test; FDR <0.05; Götz et al., 2008). Metabolic reactions were associated with metabolic pathways according to the metacyc database version 20.5, and pathways with >50% differentially expressed metabolic reactions at the level of the entire microbiome were further examined.

For the bacterial metatranscriptomes, we also determined the overall “transcriptomic activity” of each bin in each condition. This means that read pair counts were summarized for all genes of the same bin and normalized by the total number of mapping read pairs per sample. The resulting matrix was used as input for hierarchical clustering (distance: correlation; method: average) with clustvis (Metsalu & Vilo, 2015).

Finally, viral reads in the metatranscriptomes were analysed as follows: first, a de novo assembly of the remaining RNAseq reads (nonribosomal reads that did not map the host genome or the bacterial bins) was generated using metaspades version 3.15.4. Viral contigs were identified among the assembled reads using virsorter version 2.2 (Guo et al., 2021), including all predictable viral groups. To determine viral expression levels, nonribosomal RNAseq reads that did not map the host genome or the bacterial bins were remapped against the identified viral sequences using star aligner. Differentially expressed viral sequences were identified using deseq2, as described above.

### 2.7 Metabolic complementarity analyses

We used metabolic networks derived from the metagenome data and metatranscriptome data to examine the potential metabolic complementarity of the algal host and its associated microbiome. For each microbial community, a bacterium identified in the metagenome was considered active if, for at least three out of four replicates, at least 0.2% of the total bacterial transcripts mapped to the bacterium in question. For each condition (microbial community × salinity), the metabolic networks of the active bacteria were then provided to miscoto (Frioux et al., 2018) and metage2metabo (Belcour et al., 2020) to identify the metabolites predicted to be producible by the bacteria with glucose as a carbon source and the in silico added value of the bacterial metabolic capacities for the algal host. Note that Miscoto does not computationally predict actual exchanges, only how exchanges could complement the metabolic capabilities of the partners by checking whether genes (either in the algal genome or by combining algal and bacterial genomes) can be associated with all reactions that belong to the pathways involved in the production of a metabolite from the medium. In this sense, in silico added value refers to metabolites predicted to be produced by the alga only in the presence of specific precursors, which in turn are produced by the provided bacterial community.

### 2.8 Metabolite profiling by mass spectrometry coupled to gas‐chromatography

For metabolite profiling, 10 mg of freeze‐dried ground tissue was lysed (TissueLyser, 2 × 30 s, 30 MHz; Qiagen) and subsequently used to extract metabolites with 1 ml of 100% methanol (Dittami et al., 2012). After vortexing, 5 μl of ribitol (4 mM in H_2_O, >99%, Sigma‐Aldrich) were added to each sample as an internal standard before sonication (10 min at room temperature; RT). After 15 min of centrifugation (30,000 *g*, 4°C), the supernatant was recovered, evaporated under vacuum overnight, derivatized in 50 μl methoximation solution (methoxyamine hydrochloride 98% Sigma‐Aldrich, stored in a desiccator under argon at a final concentration of 20 mg/mL in pyridine), and incubated at 60°C for 1 h and afterward at RT overnight. The samples were silylated for 1 h at 40°C in 50 μl N‐Methyl‐N‐(trimethylsilyl)trifluoracetamid (MSTFA; 1 ml + 40 μl retention index mix) and centrifuged (6 min, 2500 rpm) to pellet the precipitate (Alsufyani et al., 2017). The supernatant was analysed with a 6890N gas chromatograph equipped with a 7683B autosampler (Agilent), a glass liner (Agilent, 4 × 6.3 × 78.5 mm), and a DB‐5MS column (Agilent, 30 m × 0.25 mm × 0.25 μm), coupled to a Micromass GCT Premier (Waters) time‐of‐flight mass spectrometer. The gas chromatograph was operated with helium as a mobile phase, split 10, and 250°C injector temperature. The initial oven temperature was 60°C ramping to 310°C at a rate of 15°C per min. The mass spectrometer had a source temperature of 300°C and dynamic range extension mode. The resolution was >6000 FWHM at *m*/*z* 501.97. After randomization, samples were measured twice to obtain two technical replicates. Solvent blanks were prepared and measured in parallel.

### 2.9 Analysis of metabolite data

Raw files were directly converted to the netCDF format using the DataBridge tool within the masslynx software (Waters, version 4.1), and the chromatograms were then processed with the function metaMS.runGC (version 1.0) provided within Workflow4Metabolomics (W4M) (Giacomoni et al., 2015). The metaMS package (Wehrens et al., 2014) was used to identify chromatographic peaks with the standard functions provided by XCMS. Then, the CAMERA package was used to cluster masses with similar retention times (Kuhl et al., 2012). These coeluting masses or “pseudospectra” were summarized into a final feature table in the MSP format, a format that can be used to search spectral databases. A detailed list of settings can be found in Table S2. The resulting matrix of 689 features (pseudospectra) was manually processed. To remove contaminant signals from the matrix, each peak's maximum value among all blanks was multiplied by three and subtracted from the remaining samples. Variables with less than two samples with intensities above zero and redundant ions (isotopes) were removed. The filtered data sets were then reimported into W4M for statistical analysis. Quality assessment of the data confirmed that there were no outliers and no signal drift. Data were normalized by dry weight followed by log_10_‐scaling. A *t*‐test assuming unequal variances was used to detect metabolites that were significantly different (adjusted *p*‐value < .05) in algae with each microbial community during the shift from 100% NSW to 15% NSW, and between algae with MC1 and MC2 and algae with MC3 in the 100% NSW condition. Residuals of the *t*‐test were tested for deviation from a normal distribution using the Shapiro–Wilk test (*p* < .05). In cases where significant deviations were found, they were indicated in Table S2. This was usually the case when a feature was absent in one condition. Finally, the spectra of each significant feature were compared to the GOLM libraries (Hummel et al., 2010) and an in‐house library (Kuhlisch et al., 2018) for annotation using NIST MS Search (version 2.0). Features with a reverse match score (*R*) ≥ 800 were annotated, for 700 ≤ *R* < 800, features were labelled with an additional “?”, and for 600 ≤ *R* < 700, they were marked with “??”. Clustering was carried out using clustvis as described above for the metatranscriptome data.

## 3 RESULTS

### 3.1 Selection of microbial communities

Based on screening experiments (Table S1), algae with three microbial communities were selected for the final acclimation experiments. Two of them (MC1, MC2) were based on short (3 days) treatments with antibiotics solutions, and algae with these communities could grow in diluted natural seawater medium. The last one (MC3) resulted from 5 weeks of antibiotic treatment on Petri dishes, and algae with this community were no longer freshwater‐tolerant. All three communities were dominated by different bacterial genera (Figure 1).

### 3.2 Sequencing data

**FIGURE 2:**
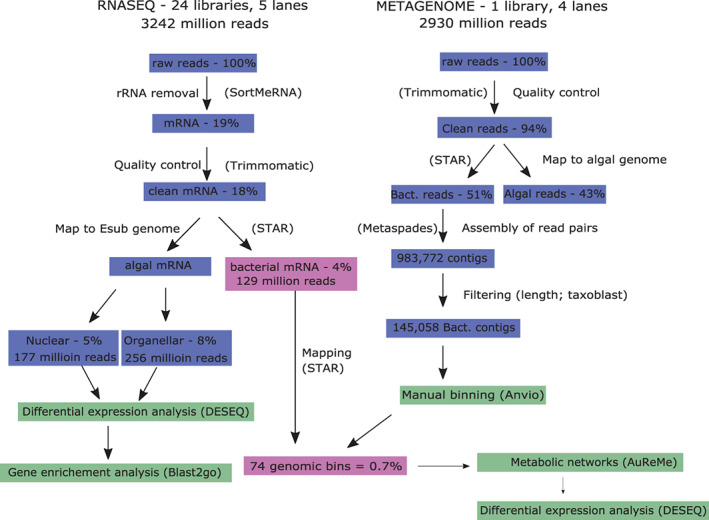
Overview of metatranscriptomic and metagenomic data obtained and the analysis pipeline. Despite extensive sequencing efforts, only 0.7% of reads mapped to bacterial genomes assembled from the metagenome. The percentages correspond to the percentage of total raw reads at the start

Illumina sequencing of four replicate cultures with each of these three microbial communities resulted in a total of 2.9 billion metagenomic reads (one library with a pool of all samples), and 3.2 billion RNAseq reads (one library per sample with an average of 135 million per library; Figure 2). Roughly half of the metagenome reads mapped with the algal genome, and the other half was considered bacterial. For the RNAseq data, despite in vitro rRNA depletion, on average, 81% of reads corresponded to ribosomal sequences, and the remaining reads mapped to the alga (both nuclear 2%–11% and organellar reads 1%–21%). Only 0.4 to 14% (5.4 million reads = 4% on average) of the total reads did not map to the algae or the ribosomal databases (see Table S3 for details) and corresponded mainly to bacterial mRNA. Viral sequences were rare, accounting for roughly 0.02% (26076) to 0.77% (1043357) of the sequences per sample. The bacterial to algal mRNA ratio varied significantly according to treatment (one‐way ANOVA, *p* < .001). It was highest in algae with MC3 in low salinity, which differed significantly from all other treatments according to a Tukey's HSD test (*p* < .001, Table S3). As sequencing data is compositional, this could be related to increased bacterial activity, decreased algal activity, or both.

### 3.3 The bacterial metagenome as a backbone for gene expression analysis

Metagenome sequencing of pooled DNA of all samples was carried out to generate a reference for the analysis of bacterial gene expression. The assembly of metagenome reads not mapping to the algal genome resulted in 145,058 contigs corresponding to 332 Mbp of sequence information. anvi'o binning of these contigs yielded 73 bins (Table S4). One additional bin was created artificially and comprised all contigs that did not fall into any other well‐defined bin (19 Mbp of sequence data). Fifty of the metagenomic bins were considered medium‐quality draft metagenome‐assembled genomes according to Bowers et al. (2017; >50% complete, <10% redundant), and among these, 35 even had a completeness ≥90% (labelled as “full”). Thirteen bins were ≥10% redundant and are thus likely to comprise genomic information from more than one organism. Most bins were taxonomically assigned to the phylum *Proteobacteria* (53), followed by *Bacteroidetes* (11), *Planctomycetes* (3), *Actinobacteria* (3), and unclassified bacteria (3). Among the phylum *Proteobacteria*, the class *Alphaproteobacteria*, specifically the orders *Rhizobiales* (12) and *Rhodobacterales* (15), were the most abundant. The phylum *Bacteroidetes* comprised six members of the genus *Flavobacteria* and one of the genus *Cytophaga*. A complete overview of the bacterial bins and assembly statistics is provided in Table S4. Each of these genomic bins was annotated, and metabolic networks were created. Together, the metabolic networks of all bacterial bins comprised 3957 different metabolic reactions (see Table S5 for summarized data; Table S6 for data separated by organism).

### 3.4 Algal gene expression

**FIGURE 3:**
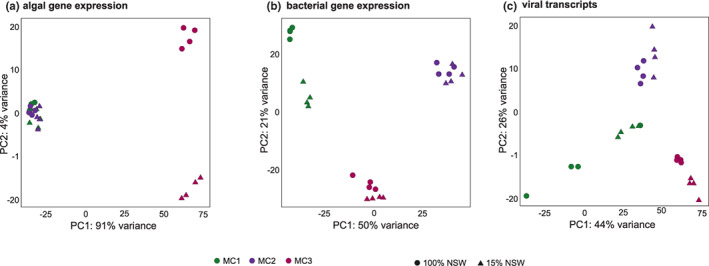
(a) Principal component analysis (PCA) of algal gene expression. (b) PCA of expression of bacterial reactions. (c) PCA of viral transcripts. Samples with MC3 differ from samples with MC1 and MC2 in all three panels, but samples with MC1 and MC2 differ mainly regarding bacterial gene expression.

**FIGURE 4:**
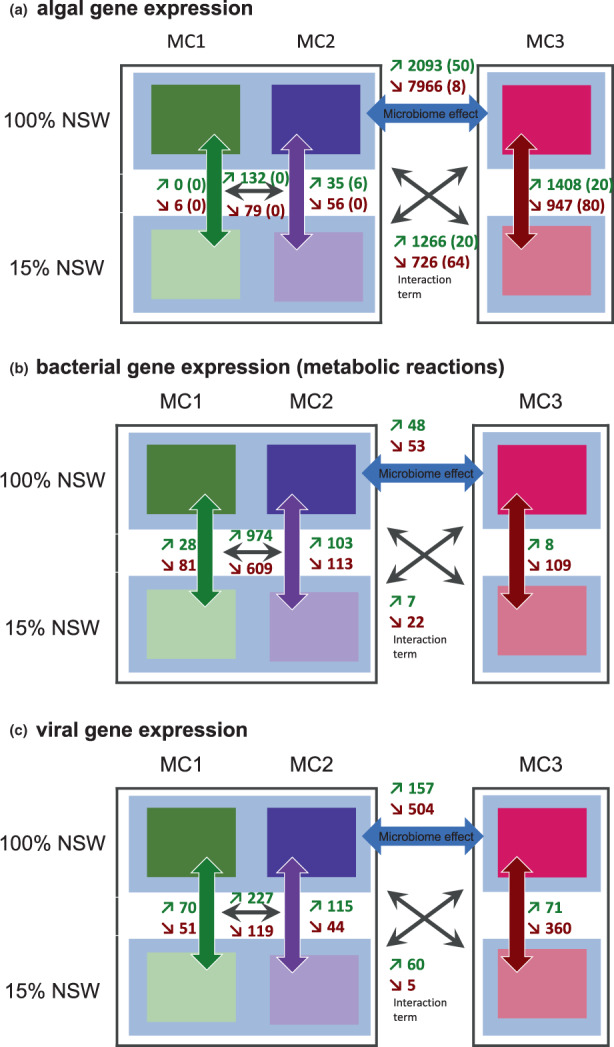
(a) Differentially expressed algal genes. The figure shows the number of differentially expressed algal genes with each of the microbial communities in 15% NSW compared to 100% NSW (MC1, MC2, and MC3); algae with MC1 + MC2 jointly compared to those with MC3 in 100% NSW (microbiome effect), the differences between algae with MC1 and MC2, and the difference in the low salinity‐response of algae with MC3 compared to those with MC1+ MC2 (interaction term, crossed arrows). Numbers in parentheses correspond to the number of overrepresented GO terms associated with the differentially regulated genes. (b) Similar analysis as A, but on the bacterial transcriptome; in this case, the analysis was based on differentially expressed metabolic reactions rather than genes. (c) Similar analysis as (a) and (b), but concerning viral sequences. Samples with MC3 exhibited the strongest host response, while the bacterial responses were equally pronounced for all three MCs

The PCA plots of algal gene expression revealed three groups (Figure 3a). The first group comprised algae with the freshwater tolerance‐conferring bacterial communities MC1 and MC2 regardless of the salinity of their culture medium. The separation of this group from the other two groups explains 91% of the variability in the data set. The other two groups correspond to the freshwater‐intolerant host‐microbiome associations in low (MC3–15%) and full salinity (MC3–100%), respectively. The separation of these two groups corresponds to 4% of the variability in the data set. DEG analyses confirmed this global pattern for algae with each microbial community. Algae associated with MC1 and MC2 exhibited only a low number of differentially expressed genes between high and low salinity conditions (6 and 91, respectively; enriched in 0 and 6 GO categories, respectively); differences in algal gene expression between the two microbial communities were moderate as well (211 significantly regulated genes in total, 0 enriched GO categories). In algae with MC3, however, 2355 genes (enriched in 100 GO terms) were differentially expressed (Figure 4a). A summary of the most important processes that were enriched among DEGs in the algae associated with the different microbial communities is given in Table 1, and details are provided in Table S7. While algae with MC3 exhibited the repression of several primary metabolic processes in 15% NSW, algae with MC1 and MC2 exhibited responses, such as the upregulation of heat shock proteins or the repression of light‐harvesting complex proteins.

**TABLE 1:** Summary of the differentially regulated processes during the response to low salinity in the algal hosts with each microbial community (MC) based on the summary of enriched GO terms from Table S7

	Downregulated in 15%	Upregulated in 15%
Algae with MC1		Heat shock protein SAM‐dependent methyltransferase dynein heavy chain protein
Algae with MC2	LHCR/LHCF Cytochrome/PSII (cp) Ribosomal proteins (cp) Transcription/Translation	C5‐epimerase heat shock protein 70
Algae with MC3	Ammonium transmembrane transport Mannose synthesis	Nitrate assimilation Photosynthesis Amino acid metabolism Vitamin biosynthesis Lipid metabolism

	Downregulated in MC3	Upregulated in MC3
Algae with MC1 + MC2 vs. algae with MC3	Photosynthesis Transmembrane transport Transcription/translation Amino acid metabolism Lipid metabolism Carbohydrate metabolism nitrogen metabolism	Cytoskeleton
Interaction term	Transmembrane transport	Amino acid metabolism Lipid metabolism Photosynthesis Vitamins Carbohydrate metabolism

*Note*: Upregulated genes were significantly induced in 15% NSW compared to 100% NSW and/or in algae with MC3 compared to algae with MC1 + 2 (interaction term). Downregulated refers to genes significantly repressed in the same conditions.

A key objective of this study was to determine how the freshwater‐intolerant algal‐microbial associations (alga + MC3) differed from the freshwater‐tolerant algal‐microbial associations (algae + MC1 or MC2), both in terms of basal gene expression in 100% NSW and regarding their response to low salinity. Regarding algal gene expression, this was accomplished by DEG analysis of the data grouping algae with MC1 + MC2 and using a two‐factor model (microbial community*salinity). In this model, algal genes significant for the factor microbial community correspond to basal differences in gene expression between algae in freshwater‐tolerant and freshwater‐intolerant algal‐bacterial communities. Overall, 10,059 genes fell in this category; 7966 were downregulated in cultures with MC3, and 2093 were upregulated in cultures with MC3. Overrepresented GO terms associated with these genes cover a range of primary metabolic and cellular processes such as transmembrane transport, GO:0055085, the amino acid catabolic process (GO:1901606), fatty acid biosynthesis (GO:0006633), or carbon utilization (GO:0015976). The processes are summarized in Table 1 and listed in more detail in Table S7.

In the same model, genes significant for the interaction term correspond to microbial community‐specific gene regulation differences in response to low salinity. Here 1266 genes were upregulated explicitly in algae with MC3 in low salinity, and 726 downregulated. The physiological processes and GO terms overrepresented among these genes are summarized in Table 1 and listed in Table S7. They comprise several typical responses to changing salinity, such as transmembrane transport (overrepresented in both up‐ and downregulated genes) or lipid metabolism with activation of lipid breakdown in algae with MC3 in low salinity.

### 3.5 Microbial gene expression

For the analysis of microbial gene expression patterns, despite the availability of a metagenome, a classical gene‐by‐gene and organism‐by‐organism approach was not feasible due to the low final read coverage and the high number of microbes/microbial genes present (only 0.7% of all reads mapped to the 73 microbial bins). Therefore, we used two alternative approaches to exploit the available data. First, we summarized gene expression data for all genes within a given metagenomic bin to determine each bin's overall transcriptomic activity. Second, we merged gene expression data for all genes predicted to catalyse the same metabolic reaction across all bins. These latter data were used to examine differences in the overall metabolic activity of the entire microbiome in the tested conditions (Table S8).

**FIGURE 5:**
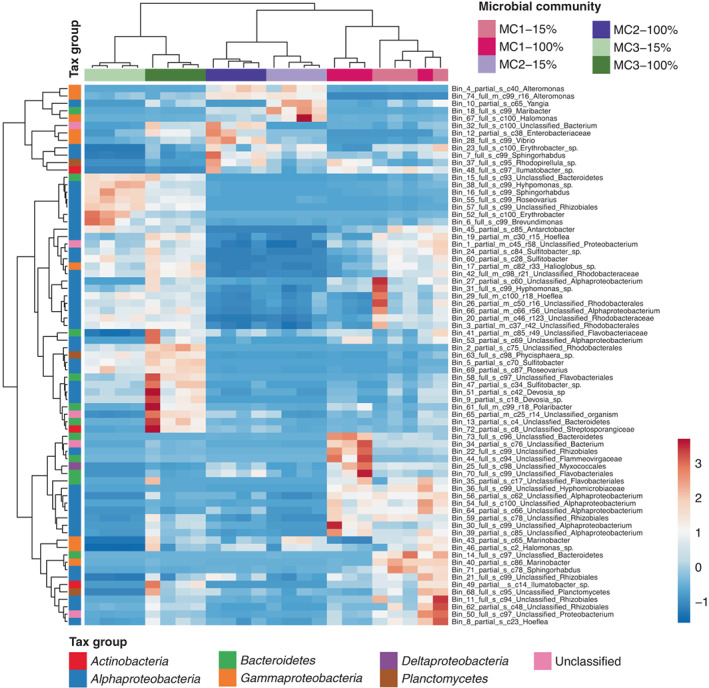
Heat map based on hierarchical clustering of normalized transcriptional activity per bacterial bin in each microbial community and condition from Table S8. The figure shows that different bacteria were active, depending on microbial community and, to a lesser extent, salinity. Clustering was based on the Pearson correlation coefficient using the average linkage method. Unit variance scaling was applied to row data, that is, for each row, the average was adjusted to 0 and the standard deviation to 1. Red indicates high relative transcriptomic activity in the given condition, and blue indicates low activity. “15%” corresponds to the treatment with 15% natural seawater (NSW), “100%” to the treatment with 100% NSW. Bins labelled “full” are predicted to be ≥90% complete, and bins labelled “partial” < 90%. The predicted completeness in % is given after the “c” in the bin name

Normalized transcriptional activity per bin was visualized in a heat map (Figure 5). While each sample contained at least one read mapping to each bin, the figure shows that each of the three microbial communities is characterized by strong transcriptomic activity of different metagenomic bins, and smaller differences within each of these clusters separate the 100% NSW from the 15% NSW conditions. A similar pattern was also observed in the PCA plot based on the microbiome's overall metabolic activity: again, “microbial community” was the main separating factor, but within each community, separation according to the salinity treatment is visible (Figure 3b), and there was variability among replicates for each community.

Expression data for the 3957 metabolic reactions (Table S5) were subjected to differential expression analysis to determine the metabolic specificities of the tested microbial communities and salinity conditions. One hundred nine significant reactions were identified in MC1, 226 in MC2, and 117 in MC3 (Figure 4b, Table S9). In MC1, gene expression differences between the salinity levels concerned, most importantly glycine biosynthesis (induced in low salinity) and quinone biosynthesis and quorum sensing (repressed in low salinity). In MC2, glycine, sorbitol, butanol, and polyamine metabolism were induced, and several genes related to nucleotide degradation and quinone metabolism were repressed in low salinity (among other reactions). Unlike for algal gene expression data, differences between MC1 and MC2 were also very pronounced, with 1583 (40%) reactions differentially expressed. In MC3, unlike in MC1 and MC2, osmolyte production (glycine–betaine, ectoine) was repressed in low salinity, along with some carbohydrate degradation pathways and ATP as well as NAD metabolism. No pathways in this microbial community contained >50% of induced reactions in low salinity, yet among the eight individual reactions was RXN‐13034 (see Table S9 for a complete list of reactions). This reaction corresponds to an oligoalginate lyase and was overexpressed in low salinity by *Alteromonas* (Bin 74), *Erythrobacter* (Bin 52), and *Sphingorhabdus* (Bin 16). Only one reaction, catalysed by a diaminobutyrate aminotransferase (R101‐RXN), was repressed in low salinity in all tested microbial communities, and no reactions were significantly upregulated in all communities. Comparing global expression patterns of MC3 with MC1 and MC2 in full salinity revealed only four pathways: hydrogen oxidation, phosphonoacetate degradation, pyruvate fermentation, and hydrogen fumarate electron transfer, all of which were upregulated in MC3.

Examining reactions significant for the interaction term, that is, microbial reactions for which the response to low salinity differed between the freshwater tolerance conferring algal‐microbial communities (MC1 and MC2) and the microbial community not conferring freshwater tolerance (MC3), only one pathway, autoinducer AI‐1 biosynthesis, emerged. This pathway was explicitly upregulated in the MC3 in low salinity, notably by bacteria of the genera *Hoeflea* (bin 29), *Roseovarius* (bins 55 and 69), and *Sulfitobacter* (bin 5). The other 21 significant reactions did not constitute pathways with >50% of genes regulated, but, given their potential importance, they were manually grouped into eight metabolic categories: ectoine synthesis, phospholipids, phosphate metabolism, selenate reduction, seleno‐amino acid biosynthesis, carbon metabolism, vitamins, and DNA repair, all of which were repressed or absent in MC3 in low salinity (Table 2). Notably, the demethylphylloquinone reduction reaction (RXN‐17007), constituting the last step of the biosynthetic pathway of vitamin K was expressed by three bins, although at low levels. Bin 32 (unclassified bacterium) expressed this reaction in samples with MC2 and MC3 in seawater, bin 19 (*Hoeflea*) expressed the reaction in samples with MC1 (both seawater and 15% NSW conditions) and samples with MC3 (seawater), and bin 67 (*Halomonas*) expressed the reaction in samples MC2 in 15% NSW. However, none of the bacteria in our metagenome expressed RXN‐17007 in MC3 in 15% NSW.

**TABLE 2:** Metabolic reactions with different low salinity responses in MC3 compared to MC1 and MC2 (interaction term).

Regulation		Taxa responsible for global profile^a^
Quorum sensing		
2.3.1.184‐RXN (acyl‐homoserine‐lactone synthase) (x6)	Induced	*Sulfitobacter* (Bin5), *Roseovarius* (Bin69, Bin55), *Hoeflea* (Bin29)
Ectoine synthesis		
R101‐RXN (diaminobutyrate aminotransferase)	Repressed	*Antarctobacter* (Bin45), *Roseovarius* (Bin55), *Halomonas* (Bin67)
R102‐RXN (diaminobutanoate acetyltransferase)	Repressed	*Antarctobacter* (Bin45), *Roseovarius* (Bin55), *Halomonas* (Bin67)
R103‐RXN (ectoine synthase)	Repressed	*Antarctobacter* (Bin45), *Roseovarius* (Bin55), *Halomonas* (Bin67)
Phospholipids		
GLYCPDIESTER‐RXN (glycerophosphoryl diester phosphodiesterase) (x3)	Repressed	*Antarctobacter* (Bin45), *Alteromonas* (Bin74), *Sphingorhabdus* (Bin16), *Sulfitobacter* (Bin5), *Erythrobacter* (Bin52), uncl. *Rhizobiales* (Bin22), *Hoeflea* (Bin29), uncl. *Bacteriodetes* (Bin73)
LIPIDXSYNTHESIS‐RXN (UDP‐2,3‐diacylglucosamine diphosphatase)	Repressed	*Hoeflea* (Bin29), *Phycisphaera* (Bin63), uncl. *Bacteroidetes* (Bin73), *Polaribacter* (Bin61), *Maribacter* (Bin 18), *Alteromonas* (Bin74), ABC
Phosphate turnover/metabolism		
3.11.1.2‐RXN (phosphonoacetate hydrolase)	Repressed	*Hoeflea* (Bin19)
ABC‐27‐RXN (phosphate ABC transporter)	Repressed	*Sphingorhabdus* (Bin16), uncl. *Rhodobacteraceae* (Bin20), *Hoeflea* (Bin29), *Antarctobacter* (Bin45), *Erythrobacter* (Bin52), uncl. *Rhizobiales* (Bin57), *Phycispheara* (Bin63), *Alteromonas* (Bin74), *Halomonas* (Bin67), *Alteromonas* (Bin4)
RXN0‐1401 (ribose 1,5‐bisphosphate phosphokinase)	Repressed	*Halomonas* (Bin67), *Hoeflea* (Bin29), uncl. *Rhizobiales* (Bin57), *Antarctobacter* (Bin45)
Selenate reduction/seleno‐amino acid biosynthesis		
RXN‐12720 (ATP‐sulfurylase) (x2)	Repressed	*Illumatobacter* (Bin48), uncl. Bacterium (Bin65), uncl. *Bacteroidetes* (Bin73), *Alteromonas* (Bin74)
RXN‐12730 (5‐methyltetrahydropteroyltri‐glutamate‐‐homocysteine S‐methyltransferase)	Repressed	*Halomonas* (Bin67)
Carbon metabolism		
RXN‐961 (ribulose bisphosphate carboxylase/oxygenase)	Repressed	Uncl. Bacterium (Bin32)
2.8.3.7‐RXN (Succinyl‐CoA‐l‐malate CoA‐transferase) (x4)	Repressed	*Sphingorhabdus* (Bin16), *Hoeflea* (Bin29, Bin19), uncl. *Rhodobacterales* (Bin2), *Hyphomonas* (Bin38), *Ilumatobacter* (Bin48)
RXN‐14365 (D‐psicose 3‐epimerase.)	Repressed	*Hoeflea* (Bin29), *Antarctcobacter* (Bin45), *Maribacter* (Bin18)
Vitamins		
RXN‐17007 (Demethylphylloquinone reductase)	Absent	*Hoeflea* (Bin19), *Halomonas* (Bin67), uncl. Bacterium (Bin32)
DNA repair		
3.1.21.2‐RXN (Endonuclease)	Repressed	*Hoeflea* (Bin19)

*Note*: The first column contains the metacyc reaction ID and, in parentheses, the corresponding enzyme. If the same enzymes carried out several (similar) reactions, these were grouped, and only one representative is shown. If different than 1, the total number of reactions catalysed by this enzyme is given in parentheses following an x after the enzyme name. “Induced” means that this reaction was more strongly upregulated in MC3 in response to 15% NSW than in MC1 and MC2. Repressed or absent means the reaction was downregulated or absent in MC3 in 15% NSW.

Abbreviation: Uncl., unclassified.

^a^ According to expression data provided per bin and reaction in Table S6.

### 3.6 Viral expression

**FIGURE 6:**
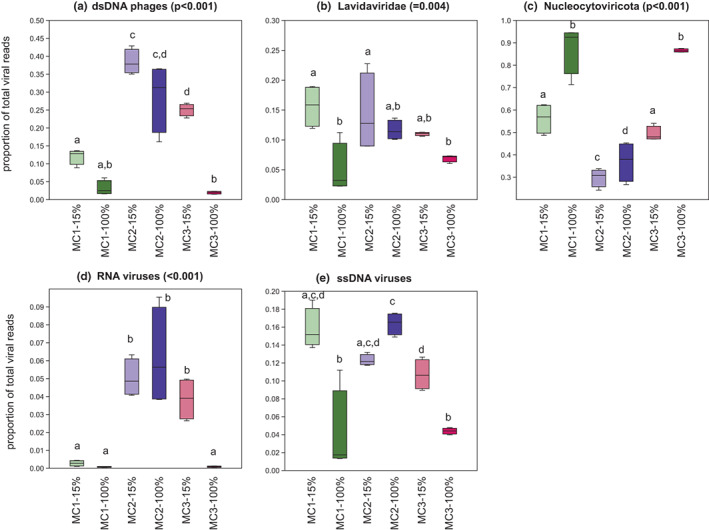
Box plot of relative abundances of viral sequences belonging to different families in all samples (MC1–MC3 = microbial community 1–3) showing the variability of the viral transcript abundance depending on microbial community and salinity. Values are given in % of the total number of viral reads per sample. *p*‐values correspond to the results of an ANOVA across all six conditions (4 replicates each). Lowercase letters indicate significant differences between treatments (Tukey's HSD test). 15% corresponds to the treatment with 15% NSW, 100% to the treatment with 100% NSW

In addition to algal and bacterial gene expression analyses, we used the generated data to search for signs of viral gene expression. Virsorter2 identified a total of 1204 viral contigs in our metatranscriptome assembly. Although these contigs represented less than 1% of the total metatranscriptome reads, the analysis of these sequences revealed marked differences in viral community composition depending on treatment and the microbial community (Figure 3c). The global patterns followed that of the microbial community data, with clear differences in viral expression depending on the community and smaller differences depending on salinity. Samples with MC1 in 100% NSW exhibited the highest relative abundance of viral sequences (Table S3), although variability between samples was high. Different viral groups were dominant depending on the microbial community and the salinity (Figure 6). For instance, reads corresponding to double‐strand DNA phages and RNA viruses were most abundant in algae with MC2. In contrast, reads corresponding to Nucleocytoviricota, large double‐strand DNA viruses that infect mainly eukaryotes were most abundant in samples with MC1 and MC3 in 15% NSW. Table S10 provides a detailed list of differentially expressed viral sequences.

### 3.7 Potential “added value” of active microbial communities

**FIGURE 7:**
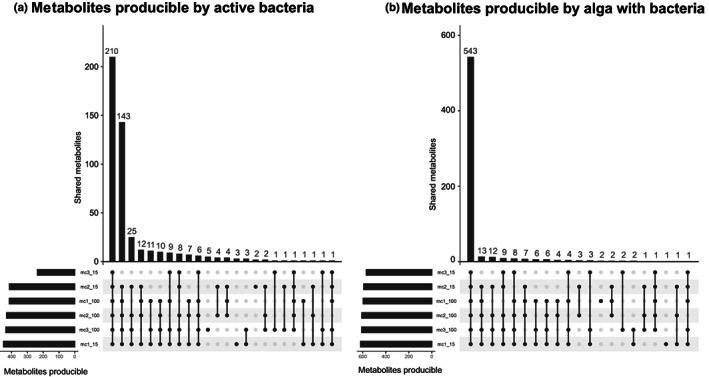
Upset plot of (a) metabolites predicted to be producible by the active bacterial communities in the different conditions, and (b) metabolites predicted to be newly producible by the algal host if given access to the metabolites producible by the bacteria. The bars represent the number of metabolites shared between the different communities and conditions. Most metabolites are producible in all communities, but MC3–15% has the lowest number of producible metabolites. A detailed list of metabolites is available in Table S11

The metabolites predicted to be producible by the bacteria active in each community and condition based on their genome‐scale metabolic networks, as well as the in silico added value of these communities for metabolite production in the host, are provided in Table S11. The communities themselves are listed in Table S12. In terms of producible bacterial compounds, metabolic networks predict between 236 metabolites for MC3–15% and 451 for MC1–15%, with MC3–15% being a clear outlier (Figure 7). When considering the in silico added value of bacterial metabolites to facilitate the production of new algal compounds, the same trend was observed, but less pronounced: again, the alga with MC3–15% was predicted to produce the fewest (595), and the alga with MC1–15% the most compounds (616). The identity of the compounds producible or missing in one or the other community/condition was variable. Regarding the compounds predicted to be produced only by bacteria, amino acids stood out: they constitute 6.3% of the compounds predicted to be produced by all communities, but 13.6% of the compounds predicted to be produced by only some communities. For the in silico added value compounds predicted to be producible by the host, aldehydes and carbohydrates were more common among the metabolites predicted to be producible only by some communities (5.6% and 23.6% of the variable metabolites, respectively vs. 2.7% and 9.7% of the metabolites predicted to be producible in all communities).

### 3.8 Metabolite profiling

**FIGURE 8:**
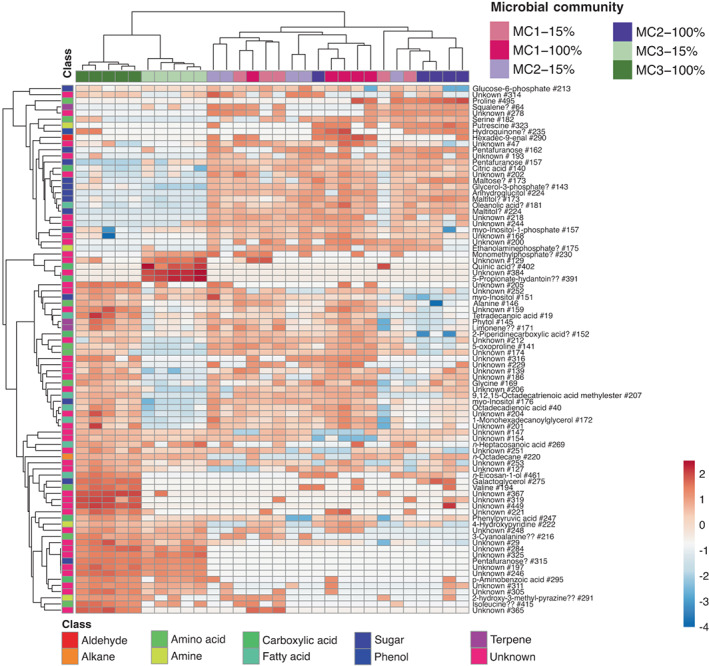
Heat map based on the abundance of each metabolite tested as significant in at least one condition. It shows clear differences in metabolite profiles obtained by GC–MS analysis depending on microbial community and for algae with MC3 depending on salinity. Clustering was based on the Pearson correlation coefficient using the average linkage method. Unit variance scaling was applied to row data, that is, for each row, the average was adjusted to 0 and the standard deviation to 1. 15% corresponds to the treatment with 15% NSW, 100% to the treatment with 100% NSW. “?” indicates features with reverse match score (*R*) < 800, “??” features with *R* < 700. Features were labelled “unknown” for *R* < 600

To complement the microbiome and transcriptome analysis, metabolite profiling was achieved by GC–MS analysis. The metabolite data set contained 609 features that were (after normalization) used for statistical testing. In total, 72 features were significantly different in at least one tested condition (Table S13). Only two features differed significantly between low and high salinity conditions for algae associated with MC1 and MC2: a feature putatively corresponding to histidinol and intermediate of the histidine biosynthesis, was downregulated in MC1 in low salinity, as well as hydroquinone in MC2 in low salinity. Cluster analysis (Figure 8) confirmed this as it did not separate these conditions. Samples with MC3, however, exhibited differences in metabolic profiles depending on the salinity, with four upregulated (quinic acid, 5‐propionate‐hydantoin, and two unknown features) and 35 downregulated features in low salinity, including a feature annotated as phytol (peak no. 145, a precursor of vitamin K) and several primary metabolites such as galactoglycerol, glycine, alanine, valine, oxoproline. For all of these, the regulation was specific to samples with MC3. Furthermore, even under control conditions (100% NSW), metabolite profiles of samples with MC3 differed from samples with MC1 and MC2, exhibiting higher abundances of 18 features (including glycerol 3‐P, pentafuranose, putrescin) and a lower abundance of 29 features (including alanine, galactoglycerol, and valine; see Figure 8). As in the other analyses, community 3 was most sensitive to changes in salinity.

## 4 DISCUSSION

This study aimed to identify mechanisms and interactions by which bacteria may impact their host's tolerance to an abiotic stressor. We addressed this question in the model brown alga *E. subulatus*, for which its ability to acclimate to fresh water depends on the associated microbiome. Three bacterial communities were created, two allowing for algal growth in low salinities, and one not supporting algal growth. One limitation of our study is that we could not actively determine the precise composition of the microbiota we generated. However, modifying the microbiota proved promising to generate hypotheses. Our data and the specific factors identified are valid only for this model system and these three specific microbial communities (and thus time points if we assume that microbiota continuously evolve). Nevertheless, we believe they provide an exciting example and consider it likely that similar mechanisms, though different compounds, may, in the future, be confirmed in other organisms.

Our data show that variations in the microbial community impacted algal gene expression profiles and metabolomic features. Freshwater‐tolerant cultures with MC1 and MC2 had fewer differentially expressed genes (Figure 4) and metabolites (Figure 8) in response to changes in salinity, suggesting that treatments were not particularly stressful for the algae with these microbial communities. On the other hand, basal expression levels, metabolite profiles, and even viral expression levels were altered in cultures with the freshwater‐intolerant bacterial community. Here, we observed a strong transcriptomic response of the host to salinity changes similar to that described by Dittami et al. (2012), where 3004 genes were significantly (*p* < .05) up‐ or downregulated in low salinity. All cultures were allowed to recover in antibiotic‐free natural seawater medium (NSW) for weeks after the initial antibiotic treatment. This was done to limit differences in the algal response to low salinity related to the direct effects of the treatments. Therefore, our hypothesis is that the microbiota is the driving factor behind the observed differences. This is in line with the results of inoculation experiments demonstrating that freshwater tolerance in antibiotic‐treated cultures has previously been restored by restoring the microbiome (Dittami et al., 2016). Still, additional direct effects from the treatment cannot be excluded with certainty.

Our data also suggest that viral transcription changes may further complexify this already complex system, as the relative abundance of viral reads varied depending on the microbial community and condition. In terrestrial plants, viruses have been shown to impact, for example, host drought tolerance by improving water balance and priming defence responses (Aguilar & Lozano‐Duran, 2022; Xu et al., 2008). Furthermore, viruses have been shown to impact the composition of microbial communities, including the alteration of virulence and biofilm formation in bacteria (Fernández et al., 2018). Tripartite trans‐kingdom interactions between eukaryotes, bacteria, and viruses have been described in mammalian guts (Pfeiffer & Virgin, 2016), corals (Thurber et al., 2017), and microalgae such as *Emiliania huxleyi* (Pollara et al., 2021), but have, to our knowledge, not been considered in macroalgal research so far. Although in our experiments, the overall viral load attached to the host and the associated microbial communities remained low (please note that viruses in the bulk aqueous were not examined), these variations affected both viruses likely to infect the host and the associated bacteria.

### 4.1 Three scenarios of microbial impacts on host stress tolerance

Here, we discuss the generated metatranscriptomic, metagenomic, and metabolomic data in the light of three different scenarios of how bacteria may impact algal freshwater tolerance. (1) Members of the microbiome of freshwater‐tolerant cultures may directly produce compounds such as osmolytes or chaperones that enhance algal freshwater tolerance or stimulate the alga to produce them. (2) The microbiome provides essential services to the alga regardless of the salinity, but under stress, the microbiome of the freshwater‐intolerant cultures may no longer provide these services, ultimately leading to a reduction in growth. (3) Under stressful conditions, the equilibrium in the microbiota may be disrupted, and certain microbes may proliferate and become harmful to the host. This phenomenon, termed dysbiosis, can be triggered both by algal and bacterial signals and possibly mitigated by the presence of other microbes or viruses.

### 4.2 No clear signs of microbial contributions to algal stress response

In the first scenario, the alga‐associated microbiome is assumed to produce compounds that enhance algal stress tolerance. In terrestrial environments, studies have highlighted, for instance, the microbial production of plant hormones, which activate plant defences, making them more resistant to pathogens, or the uptake of nutrients (e.g., via the production of siderophores that enhance the host's ability to survive in low‐nutrient environments) (Numan et al., 2018). In this context, it was shown that algae can induce siderophore biosynthesis in the freshwater bacterium (Kurth et al., 2019). When examining the low salinity response of the tested freshwater‐tolerant algal‐bacterial communities, we found little difference in algal gene expression or the metabolite profiles. Furthermore, none of the observed changes corresponded to the induction of classical stress response genes (e.g., heat shock proteins, chaperones, genes involved in synthesizing osmolytes or osmoprotectants, or transporters).

On the bacterial side, the only low‐salinity‐induced microbial pathway common to both low‐salinity‐tolerant algal‐bacterial communities was glycine synthesis. However, we did not detect significant changes in glycine concentrations in any of the tested conditions, possibly because it was further metabolized by either the bacteria or the alga. Glycine has been shown to enhance growth in some microalgae, including diatoms (Berland et al., 1979). However, no data is available on the impact of external glycine on brown algal growth rates. Furthermore, *Ectocarpus* can synthesize glycine without bacteria and does so primarily during the daytime (Gravot et al., 2010). This does not exclude the role of glycine or other bacterial compounds in the brown algal stress response, but our transcriptomic data provide little support for this hypothesis for the tested microbial communities.

An alternative way by which microbes may increase host tolerance to stressors is by priming the host and activating its “defences” even before exposure to stress – a process previously reported in kelps (Thomas et al., 2011). Indeed, a comparison of transcriptomic and metabolic profiles of algae with MC1 and MC2 versus MC3 revealed fundamental differences, indicating that the hosts, depending on the microbial community, were not in the same physiological state at the start of the experiments. However, most of the processes upregulated in freshwater‐tolerant hosts associated with MC1 and MC2 were related to the cytoskeleton and not GO categories such as “response to stimulus” or more specific subcategories. Thus, while our results cannot exclude potential defence priming effects, the algal host's transcriptomic regulation does not support this hypothesis for the tested microbial communities.

### 4.3 Loss of microbial services in intolerant cultures: Vitamin K

A second scenario is that changes in the microbiome triggered by the salinity change result in a lack of microbial services essential for the alga. Bacteria are known to provide, e.g., growth hormones to brown algae, including *Ectocarpus* (Pedersén, 1973; Tapia et al., 2016), and more profound metabolic interdependencies have been predicted based on metabolic networks (Burgunter‐Delamare et al., 2020). However, an exhaustive list of these microbial contributions is still missing. A loss of such benefits could result from shifts in the microbiome composition, changes in the activity of different microbes (Figure 5), or specific changes in bacterial gene expression patterns, possibly as a reaction to changes in algal gene expression. Our transcriptomic and metabolomic data show that the freshwater‐intolerant algae with MC3 had fundamentally different basal profiles even in seawater before applying any low salinity stress (Figure 3), and the predicted capacity of this community to produce metabolites in low salinity was reduced (Figure 7). Several algal primary metabolic processes were activated compared to algae with MC1 and MC2, such as the synthesis of amino acids and lipids, photosynthesis, carbohydrate metabolism, and transcription/translation.

In contrast, several primary metabolites were less abundant (proline, alanine, glycine, serine, citric acid, and more, Figure 6). One possible interpretation of these observations is that some bacterial functions were already absent in MC 3 in seawater but that these functions were not essential, and the algae could compensate for their absence. When transferred to low salinity, a loss of additional functions may have resulted in the observed repression of primary metabolism and reduced growth.

In addition to the coarse presence/absence view of potentially producible metabolites, our transcriptomic data highlighted bacterial metabolic processes that were less expressed in MC3 in low salinity (Table 2). Based on the *Ectocarpus* genome (Cock et al., 2010), most of these processes are probably achievable by the algal host itself without requiring input from bacteria (carbon, phosphate, selenate, phospholipid metabolism, DNA repair). This does not preclude that these compounds or activities may be helpful for the host, for instance, by reducing the metabolic cost of producing or capturing some of these compounds, thus increasing the available resources for growth. However, their provision from external sources is probably not essential.

In the same vein, ectoine synthesis was repressed in the bacterial metatranscriptome of MC3 in low salinity. Ectoine is known to serve as an osmolyte in bacteria (Czech et al., 2018), and further studies are necessary to determine if ectoine might also be released to the environment for the benefit of the host. Microalgae without an associated microbiome contain ectoine in small amounts, pointing towards a dual origin of this metabolite in the algae from their biosynthesis and from uptake (Fenizia et al., 2020). However, ectoine might be only used as an osmolyte for bacteria in 100% NSW and may no longer be required at low salinity.

Vitamin K is involved in the functioning of the photosystem in land plants. Mutants of *Arabidopsis* and *Cyanobacteria* missing the last reaction of their biosynthetic pathway are viable but exhibit increased photosensitivity (Fatihi et al., 2015). In *E. subulatus*, as in *E. siliculosus*, *Saccharina latissima*, and *Cladosiphon okamuranus* (Dittami, Corre, et al., 2020a; Nègre et al., 2019), the last step of the vitamin K biosynthetic pathway is absent from the algal metabolic network. It suggests that these algae cannot produce vitamin K independently, although this compound has previously been detected in kelps (Yu et al., 2018). If, as suggested by our transcriptomic and metabolic data, bacterial vitamin K production is repressed specifically in MC3 in low salinity, it is plausible that the resulting lack of vitamin K may negatively impact algal growth in this condition. This constitutes a promising hypothesis to be tested, for instance, via complementation experiments.

### 4.4 Indications for dysbiosis

The last of the three scenarios assumes that, in the freshwater‐intolerant algal‐bacterial associations, a change in salinity leads to a change in the bacterial community or activity (Figure 5), notably the propagation of microbial strains that harm the host (dysbiosis). Dysbiosis is a well‐studied phenomenon, especially in mammalian models. It is known to be the basis of several diseases (e.g., Hawrelak & Myers, 2004) and assumed to be widespread also in marine environments (Egan & Gardiner, 2016).

In our data, we observed a significant induction of the AI‐1 pathway, specifically in MC3 in low salinity (i.e., in the interaction term), supporting the hypothesis of the activation of the production of quorum sensing (QS) compounds. QS compounds, particularly AI‐1, have been linked to dysbiosis in several model systems. For instance, in the coral *Pocillopora*, they have been related to coral bleaching (Zhou et al., 2020). Similarly, in *Acropora*, inhibitors of AI‐1 have been shown to prevent white band disease (Certner & Vollmer, 2018). Although bacterial QS molecules may also directly impact the hosts, in several microalgae, QS molecules have been associated with the bacterial production of algicidal proteins or compounds such as proteases, amylases, quinones, or in many cases, unknown compounds (Demuez et al., 2015; Paul & Pohnert, 2011). Furthermore, we observed a four‐fold increase in the relative bacterial mRNA “activity/abundance” compared to algal mRNA reads, suggesting either a decrease in the algal transcriptomic activity or an increase in bacterial activity or abundance as typical for dysbiosis. This relative increase in transcription levels concerned primarily bacteria that were little active in the other conditions (unclassified *Bacteroidetes*, *Hyphomonas*, *Sphingorhabdus*, *Roseovarius*, unclassified *Rhizobiales*, *Erythrobacter*, *Brevundimonas* Figure 5), and the resulting community had a slightly lower potential for beneficial interactions based on our metabolic complementarity analyses.

Both observations, the induction of QS genes and the suspected increase in bacterial abundance fit well with the scenario of dysbiosis, which could be the cause or the effect of host stress. However, there are two significant limitations: First, QS compounds are not exclusively linked to dysbiosis and virulence – they may also be involved in processes such as the regulation of bacterial motility, biofilm formation, or the regulation of nutrient uptake (Zhou et al., 2016), and even resistance to bacteriophages (Høyland‐Kroghsbo et al., 2013). In our data, we did not observe the induction of virulence‐related genes. The only slight indication we have in this sense is the induction of an oligoalginate lyase reaction involved in the degradation of one of the main algal polysaccharides in MC3–15%NWS. This would be in line with a change in bacterial behaviour towards feeding on the algal host. The lack of detection of other virulence factors could be explained by the nature of our analyses, which considers only genes with known functions that are represented in the metacyc database (Caspi et al., 2018). Any (unknown) compounds or genes not represented in metacyc would not have been detected. The second limitation is linked to the first. Without further targeted experiments, it is impossible to assert if the induction of QS genes may be an indirect cause of the poor algal physiological state or, instead, if the behavioural response of the bacteria to this compound causes poor algal health. Based on these observations, we believe that the role of QS during freshwater acclimation may be of interest for future studies, for example, with QS inhibitors. These studies should also specifically examine the production of potential virulence factors to test the hypothesis of dysbiosis further.

### 4.5 Conclusion

The case of the freshwater strain of *E. subulatus* and its reliance on its microbiome for growth in fresh water is an excellent example of the importance of host‐symbiont interactions. It also demonstrates the difficulties of understanding the precise interactions in such complex systems, mainly when the models are not well‐established. Because we have not yet been able to cultivate or identify the right mix of bacteria required for freshwater tolerance, fully controlled experiments to elucidate the specific functions are not (yet) possible. As a result, we chose to modify microbiome composition empirically and then study its behaviour using a combination of metabolomics, metatranscriptomics, and metagenomics. Metabolic networks were used as a filter to combine and interpret the resulting complex data sets. This data only covers part of the biology of the organisms studied, but, in the tested microbial communities, suggested potential metabolic roles of associated bacteria in the supply of vitamin K, as well as a possible role of quorum sensing compounds in the algal‐bacterial communities that no longer exhibited growth ‐ both hypotheses that can now be tested in targeted experiments. Lastly, our findings highlight the importance of considering viruses as a potential factor influencing acclimation to environmental change by interacting with the host and the bacterial microbiome. Although these results apply only to our specific study system, we believe that such examples are valuable for comparison with other systems and will increase our awareness of and understanding of the potential ecological consequences of host–microbe interactions.

## AUTHOR CONTRIBUTIONS

Hetty KleinJan and Simon M. Dittami conceived the study with input from Catherine Boyen, Thomas Wichard, Anne Siegel, Clémence Frioux and Erwan Corre; Hetty KleinJan, Gianmaria Califano, Simon M. Dittami performed experiments; Hetty KleinJan, Méziane Aite, Simon M. Dittami, Enora Fremy, Clémence Frioux, Gianmaria Califano, Thomas Wichard performed analyses; Hetty KleinJan, Simon M. Dittami, and Clémence Frioux generated figures and tables and Hetty KleinJan and Simon M. Dittami drafted the manuscript. All authors worked on and approved of the final manuscript.

## CONFLICT OF INTEREST

The authors declare that they have no conflict of interest.

### OPEN RESEARCH BADGES

This article has earned an Open Data Badge for making publicly available the digitally‐shareable data necessary to reproduce the reported results. DNA (one library) and RNA (16 libraries) sequencing data were deposited at the European Nucleotide Archive (ENA) under project accession PRJEB43393. Metagenome assemblies, metabolic networks, and gene expression data have been deposited in the Zenodo repository (KleinJan et al., 2021, doi: https://doi.org/10.5281/zenodo.5589669).

## Supporting information


Table S1

## Data Availability

DNA (one library) and RNA (16 libraries) sequencing data have been deposited at the European Nucleotide Archive (ENA) under project accession PRJEB43393. Metagenome assemblies, metabolic networks, and gene expression data have been deposited in the Zenodo repository ([dataset] KleinJan et al., 2021). Additional data supporting this study's findings are available in the Supplementary material of this article.
